# Extensive osteonecrosis of the maxilla caused by bisphosphonates: Report of a rare case

**DOI:** 10.4317/jced.55151

**Published:** 2019-02-01

**Authors:** Murilo Santos, Karoline Silveira, Natália Souza, Davi Costa, Sirius Inaoka

**Affiliations:** 1 Resident Oral and Maxillofacial Surgery, Universitary Hospital Lauro Wanderley, Federal University of Paraiba; 2 Oral and Maxillofacial Surgeon of the department of Oral and Maxillofacial Surgery, Universitary Hospital Lauro Wanderley, Federal University of Paraiba

## Abstract

Bisphosphonates are drugs indicated for the treatment of bone metabolic diseases or malignant hypercalcemia. They are generally well-tolerated drugs, however, recent reports have described osteonecrosis of the jaw bones as a potentially serious complication related to the long-term use of these drugs. We report a case of severe osteonecrosis in a 52-years-old white woman that was taking bisphosphonates (zoledronic acid and alendronate) for the management of osteoporosis. Following a long exposure to these drugs and after being subjected to multiples exodontias, developed bisphosphonate-related osteonecrosis of the jaw compromising the whole maxilla and that extended toward the base of skull. Due to the extent of osteonecrosis, total maxillectomy and removal of all adjacent necrotic bone were planned guided by ultraviolet light. fluorescence. This case illustrates that if not treated correctly, bisphosphonate-related osteonecrosis of the jaw may cause significant morbidity, affect the quality of life and can eventually produce significant morbidity with the dissemination to noble structures and potentially causing life-threatening complications.

** Key words:**Osteonecrosis, bisphosphonate, bisphosphonate-associated osteonecrosis of the jaw, maxilla, osteoporosis.

## Introduction

Bisphosphonates are medications indicated for the treatment of bones metabolic diseases, including osteoporosis, osteogenesis imperfecta, Paget disease or for management of malignant hypercalcemia, such as those associated with multiple myeloma; cervical, lung or mammary cancer. They are generally a well-tolerated drugs, causing few side effects, such as gastrointestinal symptoms, transient low grade fever, arthralgias and increased bone pain for the injectable drugs. In addition, elevated serum levels of creatinine have been observed in some cases. However, recent reports have described osteonecrosis of the jaw bones as a potentially serious complication associated with the long-term use of these drugs (1).

This complication is more frequent in women than in men and the mean age is 66 years. The incidence of BRONJ is higher in the mandible than in the maxilla, with a 2:1 ratio (2). The criteria for the diagnosis of BRONJ include: 1 - current or previous treatment with antiresorptive or antiogenic agents; 2 - exposed bone or bone that can be probed through intra or extra oral fistula in the maxillofacial region persisting for more than 8 weeks; and 3 - no history of radiation therapy to the jaw (3). The staging of the disease is based on severity of symptoms and extension of the clinical and radiographic findings. The main objective of the treatment of BRONJ is to preserve the quality of life, controlling pain and infections and to prevent the development of new areas of necrosis (4). Treatment strategies range from conservative local wound care to aggressive resective surgery of all necrotic bones (2). Conservative strategies include systemic antibiotics, oral antibacterial rinse, and debridement of loose necrotic bone. Recent studies have demonstrated to be promising the use of non-surgical therapeutic strategies such as platelet rich plasma (5), parathyroid hormone (6), hyperbaric chamber (7).

The aim of the present study is to present an atypical case of BRONJ that, due to its severity it was necessary to perform total maxilectomy.

## Case Report

A was 52-years-old white woman with history of hypertension, diabetes, rheumatoid arthritis treated with corticoid (prednisone 10mg/day) was evaluated in our department with complains of recurrent infections and maxillary bone exposure. The patient reported that from 2007 started treatment for osteoporosis with zoledronic acid (5mg/month) that was replaced after five years by oral alendronate sodium (70mg / week). In 2013 underwent multiple exodontias and after 10 months developed persistent pain, recurrent infections and bone exposure at the sites of extractions. The patient was treated with multiple antibiotics and curettage by her general dental practitioner. In 2016, after worsening of the condition, she was referred to the Department of Oral and Maxillofacial Surgery of Universitary Hospital (Federal University of Paraiba, Brazil).

**Figure 1:**
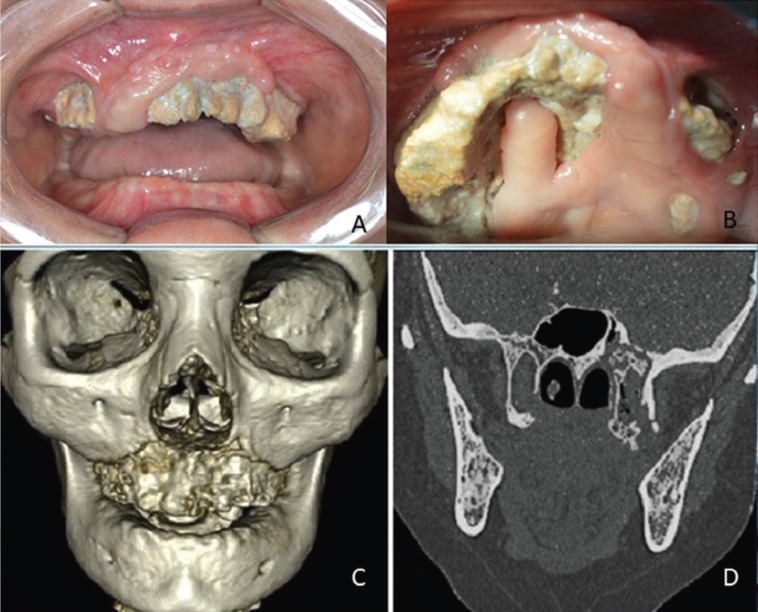
A: Intra oral clinical picture of extensive exposed necrotic bone. B: Palatine view showing exposed necrotic bone. C: Three-dimensional reconstruction CT-scan showing necrotic area compromising the whole maxilla. D: Coronal bone window image showing necrotic bone extended toward the skull base.

The clinical examination revealed edema and erythema in the third middle of the face, total maxillary edentulism, presence of necrotic bone exposure involving an extensive area of the maxilla associated with dehiscence in the alveolar and palatine mucosa on the left side and minor points on the right side (Fig. 1A,B) which was notorious for its fetid odor and spontaneous purulent drainage. A CT-scan revealed necrotic bone widen throughout all maxilla and the left pterygoid process extending the proximity of skull base. A three-dimensional reconstruction view allowed clear visualization of the separation line at the Le Fort I level (Fig. 1 C,D).

**Figure 2:**
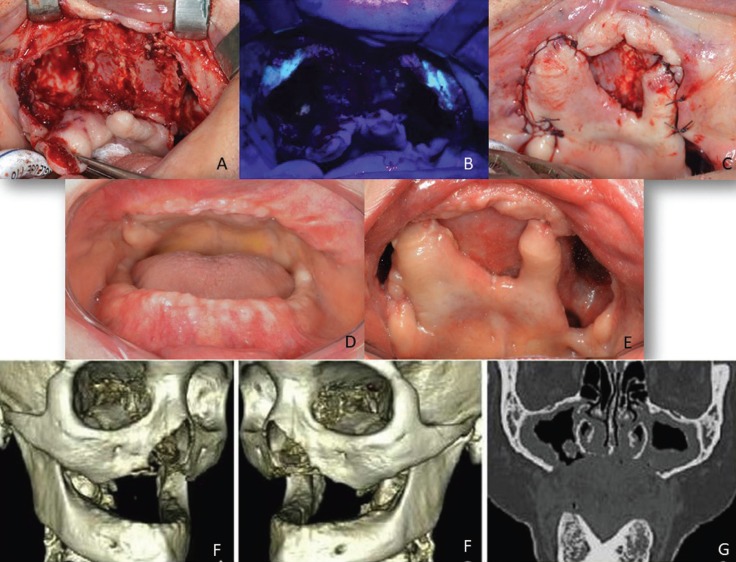
A: Clinical pictures of transoperative after removal necrotic bone. B: Fluorescence view after removal necrotic bone. C: Suture of the residual soft tissue. D: Six-months postoperative intra oral clinical picture. E: Palatine view showing oro-antral comunication. F: sex months postoperative three-dimensional reconstruction CT- scan showing absence of necrotic bone. G: six months postoperative Coronal bone window view.

The initial management included treatment for infection and the acute pain. The patient was given Amoxicillin 500mg plus Clavulanic Acid 125mg and Paracetamol 500mg as well as mouthwash (chlorhexidine digluconate 0.12%). Subsequently, due to the extent of osteonecrosis, total maxillectomy and removal of all adjacent necrotic bone were planned. Bone resection was guided by ultraviolet light fluorescence through the use of 100mg doxycycline twice daily for 10 days prior to surgery (Fig. 2A-C). In the immediate postoperative period, the patient was fed by nasal enteral tube to avoid food residues at the surgery site. In addition, smeared gauze with antibiotic ointment (bacitracin and neomycin) changed daily in the first seven days was utilized. Antibiotic therapy was maintained until 10 days after surgery and treatment with Tocopherol (500 mg, twice a day) and Pentoxifylline (400 mg, twice a day) was started aiming at reducing fibrosis and stimulating local vascularization for a period of eight months.

**Figure 3:**
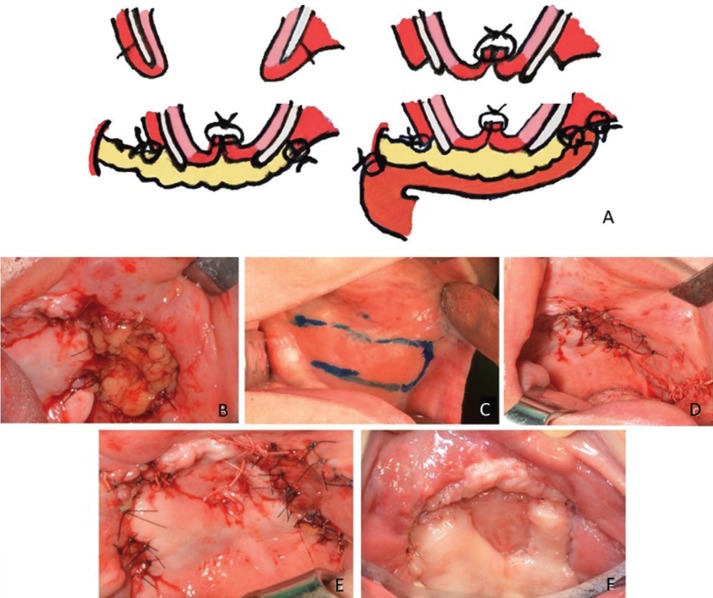
A: The drawing illustrating the sequence of the closure of oro-antral communication in three layers: the surrounding oral mucosa to form the floor of the maxillary sinus, buccal fat pad as pedicled graft and myomucosal flap from the buccinator muscle. B: Bucal fat pad as pedicled graft used to close oro-antral comunication. C: Myomucosal flap drawing. D: Appearance after tunneling myomucosal flap from the buccinator muscle suture . E: Immediately postoperative clinical appearance. F: Twelve months postoperative showing absence of oro-antral communication and exposed bone.

After a period of Six-months, complete tissue healing was observed (Fig. 2D,E) Postoperative CT-scan performed six-months after the first surgery revealed satisfactory healing of the remaining bone (Fig. 2F,G) and then closure of the oro-antral communications left by bone resection was realized, as there was not sufficient tissue for primary closure. Closing was performed in three layers (Fig. 3A). The first layer consisted of the surrounding oral mucosa to form the floor of the maxillary sinus, followed by a layer of the buccal fat pad as pedicled graft and covered as a tunneling myomucosal flap from the buccinator muscle (Fig. 3B-F). Currently, the patient is without pain nor signs of relapse.

## Discussion

BRONJ most commonly affects women, due to cases involving treatments for breast cancer and osteoporosis and the mean age is 68 years (3,8). The risk of developing BRONJ is greater with the use of intravenous bisphosphonates in cancer patients, the most common malignant diseases are breast cancer and multiple myeloma, and was first reported with use of zoledronic acid and disodium paminodrate, however, oral bisphosphonates for the treatment of osteoporosis can also cause BRONJ. Despite the lower incidence, the number of cases is significant and probably underestimated (3,8,9). Our patient was female and used intravenous zoledronic acid monthly for five years due osteoporosis and was subsequently replaced with oral alendronate.

BRONJ can be misdiagnosed as alveolar osteitis, sinusitis, gingivitis / periodontitis, periapical pathologies, fibro-osseous lesion, sarcoma and chronic sclerosing osteomyelitis (3). In this case, the patient remained on non-effective treatments for two years, probably being treated wrongly as some of these conditions. The delay in providing timely and proper treatment led to the need of performing total maxillectomy causing high morbidity.

There are rare cases of spontaneous development BRONJ, most cases affect patients who underwent dentoalveolar surgeries. Therefore, patients and practitioners should be advised about the relation of treatment with bisphosphonates and the risk of developing BRONJ. Patients who will start bisphosphonate treatment, if medical conditions allow it, should delay the treatment until oral health is optimized to minimize the risks of developing BRONJ, this decision must be made together with the patient´s physician (10).

The incidence of BRONJ depends on the type of medication used, duration of administration, and medical conditions such as the presence of systemic diseases, associated medication and local lesions. The patient in this report had multiple systemic diseases such as diabetes mellitus, rheumatoid arthritis and was also using corticosteroids and various studies have reported that the use of corticosteroids, immunosuppressive therapies and diabetes mellitus are considered important risk factors for the development of this complication (11).

The extent of bone resection, in most cases, is subjective and depends on the transoperative parameters. Characteristics that do not correlate with bone vitality, such as coloration, bone bleeding and others, are observed. As a way to allow surgical standardization, fluorescence-guided bone resection has emerged and is a technique that allows the surgeon to distinguish between viable and necrotic bone, so there is no other technique that allows visualization of residual necrotic bone (12). The fluorescence technique to guide the bone resection was effective in this case, because it allowed the total removal of the necrotic bone tissue, as it can be visualized on the postoperative CT-scan.

In the case presented here, due to dehiscence caused by osteonecrosis and cicatricial retraction, the patient develops oro-antral communications. A treatment plan was established with the use of three layers: 1 - Surrounding oral mucosa; 2 - Buccal fat pad as pedicled graft and 3 - Myomucosal tunneling flap from the buccinator muscle. The buccal fat pad has been proved to be a low risk approach and is associated with high success rate, including for the closing of large oral communications like this one (13). Furthermore, the myomucosal buccinators flap are also an excellent option to reconstruc palatal defects, these are functionally and aesthetically good, with few failures and low morbidity at the donor site (14).

Pentoxifylline and Tocopherol were used in this case in order to improve vascularization, accelerate healing and to avoid fibrosis of adjacent soft tissues. Pentoxifylline is used in the management of vascular diseases, such as ischemic heart disease and intermittent claudication, improves peripheral blood flow, increasing flexibility and vasodilation of erythrocytes. It also inhibits tumor necrosis factor alpha and may decrease inflammatory reactions and fibrosis. It is also very likely to have an effect on the decrease of pain and the acceleration of scarring of radiation-induced soft tissue necrosis. Tocopherol reduces oxygen free radicals, its antioxidant properties occur through the inhibition of platelet aggregation and also reduces tissue fibrosis. The combination of these two medications is well tolerated and has shown efficacy in the treatment of BRONJ (15).

## Conclusions

We report an atypical case of BRONJ extending across the maxilla and getting close to skull base. If not treated correctly, this pathology can evolve severely to affect the quality of life and can eventually produce significant morbidity with the dissemination to noble structures and potentially causing life-threatening complications.
